# CK2-mediated CCDC106 phosphorylation is required for p53 degradation in cancer progression

**DOI:** 10.1186/s13046-019-1137-8

**Published:** 2019-03-18

**Authors:** Yichong Ning, Chunqing Wang, Xin Liu, Yan Du, Shunlian Liu, Kaili Liu, Jianlin Zhou, Chang Zhou

**Affiliations:** 1 State Key Laboratory of Developmental Biology of Freshwater Fish, College of Life Science, Hunan Normal University, Changsha, 410081 Hunan China; 2 Key Laboratory of Protein Chemistry and Developmental Biology of the Ministry of Education, College of Life Science, Hunan Normal University, Changsha, 410081 Hunan China; 3 Present address: Department of Biochemistry and Molecular Biology, University of New Mexico Health Sciences Center, Albuquerque, NM 87131 USA; 4 Present address: Reproductive & Genetic Hospital CITIC-XIANGYA, Human, Changsha, 410008 China

**Keywords:** Protein kinase CK2, CCDC106, p53, Breast cancer, Cervical cancer

## Abstract

**Background:**

Dysfunction of p53 is a key cause of cancer development, while CCDC106 can reduce p53 stability and is associated with lung cancer. However, the roles of CCDC106 in other cancer types and its upstream regulators have not been investigated.

**Methods:**

The phosphorylation status was investigated by in vitro kinase assay and Western blotting using phosphorylation-specific antibodies. Co-immunoprecipitation assay and GST-pulldown were used to detect protein interaction. Cell viability, apoptosis, colony formation, wound-healing and invasion assays were measured for in vitro functional analyses. The in vivo effect of CCDC106 on tumor growth was investigated using a subcutaneous xenograft tumor mouse model.

**Results:**

We demonstrated that CCDC106 knockdown enhanced apoptosis by stabilizing p53 and suppressed cell viability, colony formation, migration and invasion in cervical cancer HeLa and breast cancer MCF7 cells with wild-type p53 (wtp53), whereas CCDC106 overexpression exerted the opposite effects in normal breast epithelial HBL100 and cervical cancer SiHa cells with wtp53. However, CCDC106 had no similar effects on p53-mutant cervical and breast cancer cells (C33A and MDA-MB-231). Further study showed that CK2 interacts with CCDC106 through its regulatory β subunit and then phosphorylates CCDC106 at Ser-130 and Ser-147. The phosphorylation of CCDC106 at Ser-130 and Ser-147 is required for its interaction with p53 and nuclear localization, respectively. Inhibiting CCDC106 phosphorylation by substituting both Ser-130 and Ser-147 with alanine or treating cells with the CK2 inhibitor CX-4945 abrogated CCDC106-induced p53 degradation and its oncogenic function in cells with wtp53. Wildtype CCDC106, but not Ser-130/− 147 mutant CCDC106, enhanced tumor growth and p53 degradation in a xenograft mouse model. Moreover, suppression of CCDC106 increased CX-4945 sensitivity of cancer cells with wtp53.

**Conclusion:**

This study revealed a CK2/CCDC106/p53 signaling axis in the progression of breast and cervical cancers, which may provide a new therapeutic target for cancer treatment.

**Electronic supplementary material:**

The online version of this article (10.1186/s13046-019-1137-8) contains supplementary material, which is available to authorized users.

## Background

The p53 tumor suppressor has been referred to as the “guardian of the genome” and “policeman of the oncogenes” because of its vital role in sensing and reacting to DNA damage and oncogenic signaling [1]. Dysfunction of p53 is a key cause of cancer development. Dysfunction of p53 can result from mutation of the p53 genomic locus or dysregulation of p53 activity [2]. Mutation of the p53 gene is the most frequent event in human cancers and occurs in approximately half of all cancer cases [3]. However, the frequency of p53 mutation is much lower in breast (~ 30%) and cervical (<5%) cancers [3–5]. In tumors that retain wild-type p53 (wtp53), p53 is often inactivated by upregulating the negative regulators or downregulating the positive regulators of p53 [6]. MDM2 and MDMX, two main negative regulators of p53, have been shown to be overexpressed in 38 and 65% of breast cancer cases, respectively [7], whereas in most cervical cancers, wtp53 is inactivated by the human papillomavirus (HPV) E6 oncoprotein [8]. We previously reported that the CCDC106 protein interacts with the p53 protein and promotes the degradation of p53 [9], suggesting that CCDC106 is likely involved in cancer progression. Recently, Zhang X et al. showed that CCDC106 promotes nonsmall cell lung cancer (NSCLC) cell proliferation, and its expression is significantly correlated with advanced TNM stage, positive regional lymph node metastasis, and poor overall survival in 183 NSCLC cases [10]. However, the roles and upstream regulators of CCDC106 have not been investigated in other cancer types.

Protein kinase CK2 (formerly known as casein kinase II) is a conserved serine/threonine kinase. It often exists as a heterotetramer consisting of two catalytic subunits (CK2α and/or CK2α′) and two regulatory subunits (CK2β) [11]. CK2α and CK2α′ are two closely related catalytic isoforms that both have catalytic activity in the presence or absence of CK2β. CK2β is not essential for catalytic activity but contributes to the recognition of a specific substrate [12]. CK2 is a constitutively active enzyme with a variety of substrates and has been implicated in a diverse array of biological processes [13, 14]. Elevated CK2 expression has been observed in breast, cervical and other cancers and is associated with a poor prognosis [15, 16]. CK2 inhibitors, such as CX-4945 and CIGB-300, have been demonstrated to be promising therapeutic agents against various cancers, including breast [17] and cervical cancers [18]. CK2 has been reported to be involved in dysregulation of the p53 pathway. It can directly phosphorylate p53 and then inhibit the DNA binding activity of p53 [19]. Additionally, CK2 can indirectly affect p53 activity by phosphorylating the regulators of p53. For example, CK2 phosphorylates MDM2 at S260 and S269 and then increases MDM2-mediated degradation of p53 and suppresses the p53-dependent cell cycle [20]. CK2 also reduces p53 stability by phosphorylating and activating SIRT1, which deacetylates p53 [21].

In this study, we demonstrated that CCDC106 inhibits p53-mediated apoptosis, leading to cancer progression. CK2 phosphorylates CCDC106 at Ser130 (S130) and Ser147 (S147) and then stimulates its interaction with p53 and CCDC106-mediated degradation of p53. Knockdown of CCDC106 or inhibition of CCDC106 phosphorylation suppresses the progression of breast and cervical cancers.

## Methods

### Plasmids, siRNAs and antibodies

The expression plasmids HA-p53, Myc-CCDC106, EGFP-CCDC106, Myc-CK2α and HA-CK2β have been described previously [9, 22]. Site-directed mutagenesis was performed by overlap extension PCR to generate three CCDC106 mutants (S130A, S147A and S130/147A, where Ser-130, Ser-147 and both Ser-130 and Ser-47 were substituted to Ala, respectively). To construct EGFP-tagged and GST-tagged expression plasmids, the inserts of the above Myc-tagged CCDC106 expression plasmids were subcloned into the pEGFP-C3 vector at EcoRI/KpnI sites and the pGEX-4 T-2 vector at EcoRI/NotI sites. siRNAs against the CCDC106 gene and CK2β have been validated by our group [9] and Garin D et al. [23], respectively. Bax, Bcl2, phospho-CCDC106 (S130) and phospho-CCDC106 (S147) antibodies were purchased from ABclonal Technology (Wuhan, China). The p53 and CCDC106 antibodies were obtained from Cell Signaling Technology (Danvers, MA, USA) and Signalway Antibody Co., Ltd. (Nanjing, China), respectively.

### Cell culture and transfection

The human embryonic kidney cell line HEK293, breast cell lines (HBL100, MCF-7, and MDA-MB-231) and cervical cancer cell lines (HeLa, CaSki, C-33A, and SiHa) were purchased from ATCC (Manassas, VA, USA). Cells were cultured in DMEM or RPMI 1640 supplemented with 10% fetal bovine serum, 100 U/ml penicillin, 100 μg/ml streptomycin, and 2 mM L-glutamine at 37 °C in a 5% CO_2_ incubator. Transfection was carried out using Lipofectamine 2000 according to the manufacturer’s instructions (Invitrogen, Carlsbad, CA, USA).

### Bacterial expression and purification of recombinant proteins

The GST-tagged CCDC106 or p53 expression plasmids were transformed into the *E. coli* strain BL21. The transformants were grown at 37 °C until an OD600 of 0.5–0.6 was reached. A final concentration of 1 mmol/L IPTG was then added to induce the expression of GST-fusion proteins for 6 h at 30 °C. GST fusion proteins were purified using glutathione agarose (Pierce, Rockford, IL, USA).

### In vitro phosphorylation assay and Phos-tag SDS-PAGE

The purified GST-fusion proteins (GST-CCDC106, GST-S130A, GST-S147A and GST-S130/147A) were incubated with recombinant CK2 holoenzyme (New England Biolabs, Ipswich, MA, USA) in CK2 reaction buffer supplemented with 200 μM ATP at 30 °C for 1 h. Then, the reaction mixture was separated by Phos-tag SDS-PAGE (Wako, Osaka, Japan) and transferred to PVDF membranes (Bio-Rad, Hercules, CA, USA). The membranes were incubated with anti-GST antibody and HRP-conjugated secondary antibody. In the gel with Phos-tag, the phosphorylated proteins migrate slower than their corresponding dephosphorylated counterparts [24].

### GST pull-down assay

A GST pull-down assay was performed as described previously [25]. HEK293 cells were transfected with Myc-CCDC106, Myc-S130A, Myc-S147A or Myc-S130/147A. At 24 h posttransfection, cell lysates were harvested, treated without or with λ-phosphatase (New England Biolabs), and then incubated with bacterially expressed and purified GST-p53 fusion protein. GST-p53 was pulled down with glutathione agarose beads, and the associated Myc-CCDC106 fusion protein was analyzed by Western blotting with an antibody against Myc-tag.

### Coimmunoprecipitation (co-IP) assay

Co-IP was performed as described previously [26]. For the co-IP of transiently expressed proteins, HEK293 cells were cotransfected with HA-CK2β and Myc-CCDC106 and harvested at 24 h posttransfection. Cell lysates were prepared and immunoprecipitated with rabbit anti-Myc antibody or preimmune rabbit IgG, and the precipitated proteins were analyzed by Western blot analysis using murine anti-Myc and anti-HA antibodies. For co-IP of endogenous CCDC106 and CK2β proteins, lysates of HeLa cells were immunoprecipitated with murine anti-CK2β or preimmune murine IgG, and the precipitated proteins were analyzed by Western blot analysis using rabbit anti-CCDC106 and anti-CK2β antibodies.

### Subcellular localization analysis by fluorescence microscopy

To analyze the localization of EGFP fusion proteins, HeLa cells were transfected with individual EGFP fusion protein expression plasmids. At 24 h posttransfection, the cells were fixed, and fluorescent signals were observed under a fluorescence microscope (Axioskop 2, Carl Zeiss, Germany). To analyze the localization of endogenous CK2β and CCDC106 protein expression, HeLa or MCF7 cells were fixed and sequentially incubated with primary antibodies (murine anti-CK2β monoclonal antibody and rabbit anti-CCDC106 polyclonal antibody) and secondary antibodies Texas Red-conjugated anti-mouse IgG (red) and FITC-conjugated anti-rabbit IgG). Nuclei were stained with Hoechst 33258 (blue).

### Generation of stable cell lines

Lentiviral particles expressing CCDC106, S130/147A, or empty vector were purchased from GeneChem (Shanghai, China). Lentiviral particles expressing CCDC106 shRNA or scrambled shRNA were obtained from GeneCopoeia (Guangzhou, China). The target sequences for the design of CCDC106 shRNA have been described previously [9]. Cells were infected with lentiviral particles and selected with puromycin.

### Apoptosis assay

Cells were sequentially stained with annexin V-FITC and propidium iodide (PI) (Invitrogen, Carlsbad, CA, USA) and then analyzed by CytoFlex flow cytometer (FCM) (Beckman Coulter, Brea CA, USA).

### MTT assay

Cells were seeded in serum-free medium for 24 h and then seeded in 96-well plates with normal medium for the indicated times. Cell viability was determined by an MTT assay as described previously [26].

### CX-4945 sensitivity

Cells were seeded on 96-well plates and treated with different concentrations of CX-4945 (Selleck Chemicals, Houston, TX, USA) for 24 h. Then, cell viability was determined by an MTT assay. Based on the results of the MTT assay, the IC50 (half maximal inhibitory concentration) of CX-4945 was calculated using Microsoft Excel.

### Colony formation assay

Cells were cultured in 6-well plates until colonies became visible to the naked eye. The colonies were subsequently rinsed with PBS, fixed with methanol and stained with Giemsa.

### Wound-healing and invasion assays

For wound healing assays, cells were cultured in a 24-well plate until they reached 90% confluency and were subsequently serum-starved overnight. After scratching, the cells were cultured in medium with a low concentration of serum (1%) until complete closure of the scratch wound was observed. Images were obtained at different timepoints using an inverted microscope. Invasion assays were performed using a Corning Matrigel invasion chamber (Tewksbury, MA, USA), which consisted of a cell culture insert with an 8 μm pore size PET membrane uniformly coated with Matrigel matrix.

### Xenograft tumor model

BALB/c nude mice (female, 40–44 days old) were purchased from SLACCAS Jingda (Changsha, China). A total of 1 × 10^7^ cells were injected into the backs of nude mice. The length and width of the tumors were measured by a Vernier caliper every 3 d, and the tumor volume was calculated by the following formula: volume = width^2^ × length / 2. The experiments were approved by the Animal Care and Use Committee of Hunan Normal University, and all animals were handled in accordance with the guidelines of the Hunan Provincial Council on Animal Care.

### Statistical analysis

All statistical analyses were performed with Excel 2010 software (Microsoft, Seattle, WA, USA). The values are presented as the mean ± S.D. Differences between two groups were analyzed with Student’s t-test. The level of statistical significance is expressed as a *p*-value; *, *p*-value ≤0.05 and **, *p*-value ≤0.01.

## Results

### Inhibition of CCDC106 stabilizes p53 protein and suppresses cell viability, migration and invasion in cancer cells with p53

**Fig. 1:**
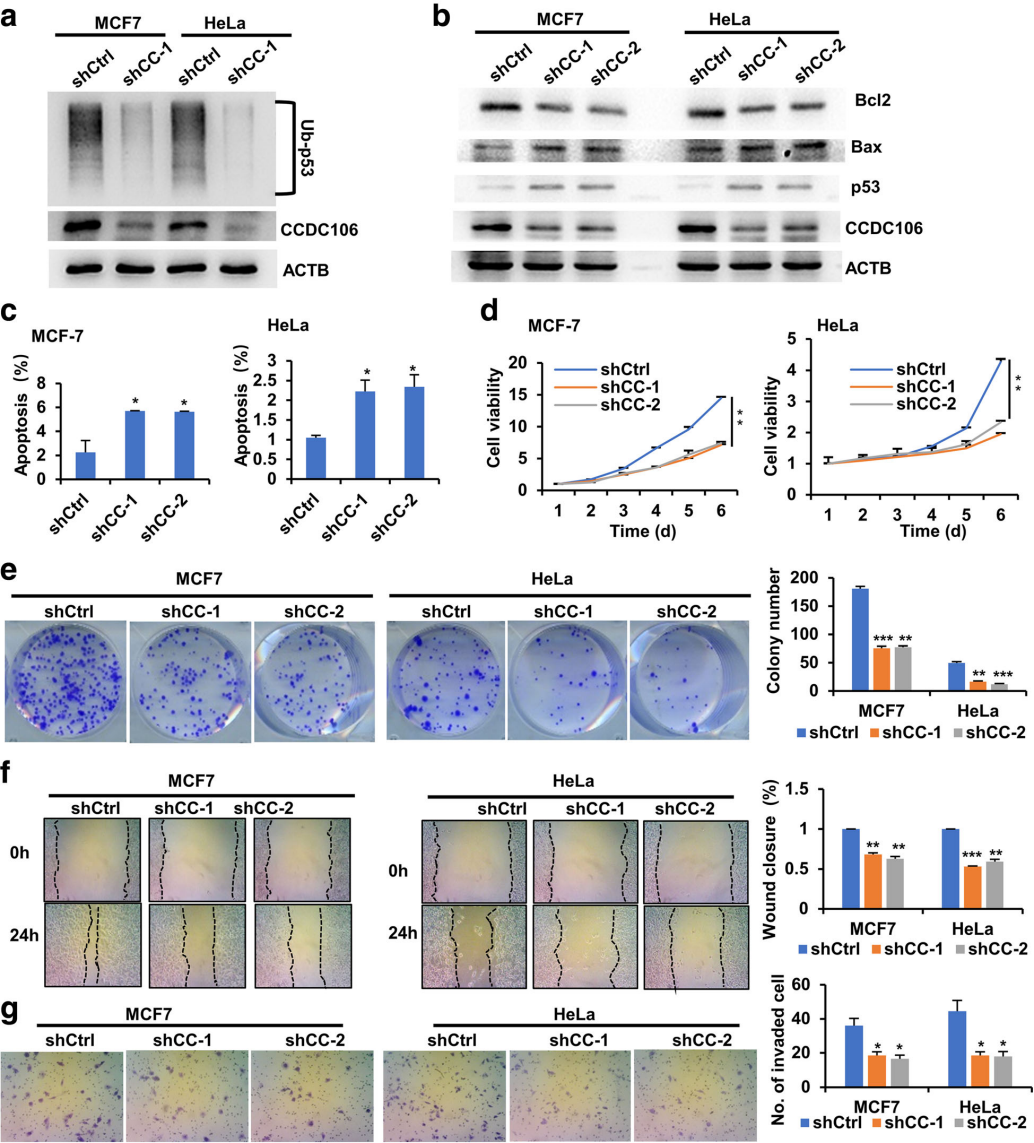
Knockdown of CCDC106 stabilizes p53 and inhibits cell growth, migration and invasion of cancer cells. **a** Ubiquitination status of p53 in MCF7 and HeLa cells stably expressing scrambled shRNA (shCtrl) or CCDC106 shRNA-1(shCC-1). Cells were treated with MG132 for 4 h, and cell lysates were then prepared and immunoprecipitated with an anti-p53 antibody. The precipitated complexes were subjected to Western blotting (WB) using an anti-ubiquitin antibody to detect ubiquitinated p53 (Ub-p53). **b** The influence of CCDC106 knockdown on the levels of p53 and its targets. **c** The apoptosis rates were investigated by annexin V-FITC/PI double staining and FCM analysis (FCM graphs are shown in Additional file 1: Figure S1b). **d** Cell viability was determined by an MTT assay. **e** Representative images of cell colonies (left panel) and statistical analysis of the cell colony numbers (right panel). **f** Representative images (left panel) of a wound area at the indicated timepoint and the percentage of wound closure at 24 h after scratching (right panel). **g** Representative images (left panel) and statistical analysis (right panel) of invaded cells in the Transwell invasion assay. All the values are presented as the means ± S.D. for at least three independent experiments. Differences between control and experimental groups were analyzed by Student’s t-test; *p-value ≤0.05, **p-value ≤0.01, and ***p-value ≤0.001

Based on our previous study [9], CCDC106 reduces p53 stability, while MG132 can suppress CCDC106-stimulated degradation of p53. In this study, we further showed that CCDC106 depletion reduced the polyubiquitination of the p53 protein in MCF7 and HeLa cells with wtp53 and high levels of endogenous CCDC106 protein (Fig. 1a, Additional file 1: Figure S1a). These results indicated that CCDC106 promotes the degradation of p53 in a ubiquitin proteasome-dependent manner.

The p53 tumor suppressor mainly functions as a transcription factor and has been reported to be able to upregulate Bax transcription but downregulate Bcl2 transcription [27]. Therefore, we investigated the influence of CCDC106 knockdown on the levels of p53 and its downstream targets (Bax and Bcl2) in cancer cells (MCF7 and HeLa) harboring high expression of CCDC106. As expected, suppression of CCDC106 increased the expression levels of p53 and Bax while decreasing the expression levels of Bcl2 (Fig. 1b). Bcl2 and Bax are responsible for inhibiting and inducing apoptosis, respectively [28]. Therefore, we analyzed the influence of CCDC106 on apoptosis and cell viability. The results showed that knockdown of CCDC106 increased apoptosis (Fig. 1c, Additional file 1: Figure S1b) but reduced cell viability in both HeLa and MCF7 cells (Fig. 1d). Furthermore, knockdown of CCDC106 inhibited colony formation, migration and invasion of both HeLa and MCF7 cells (Fig. 1e-g). However, suppression of CCDC106 had no significant effects on the stability of mtp53 and apoptosis and biological behaviors in C33A cells with mtp53 (Additional file 1: Figure S1b and S2).

### Protein kinase CK2 phosphorylates CCDC106

**Fig. 2:**
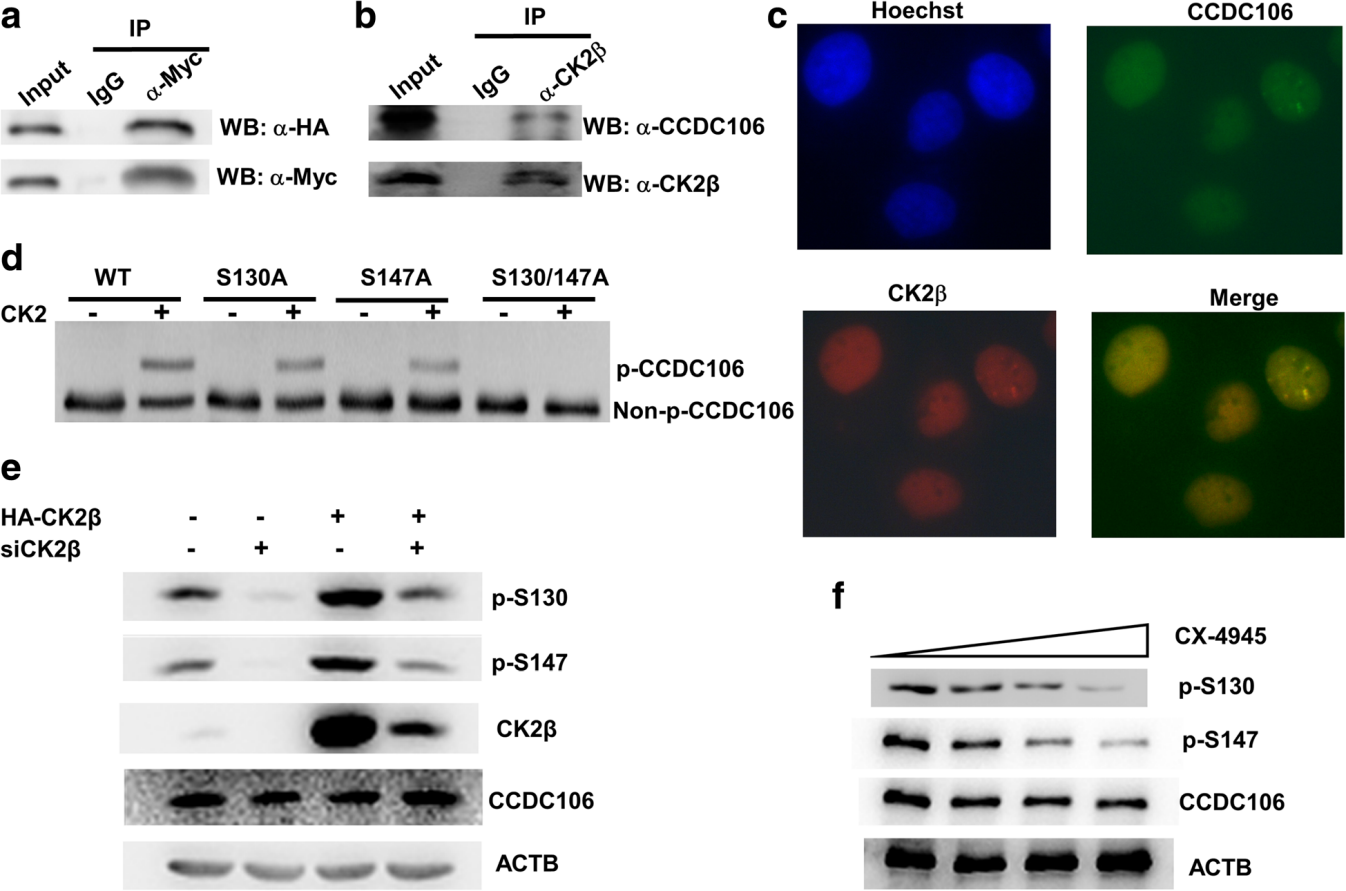
CK2 interacts with and phosphorylates CCDC106. **a** and **b** The interaction between CK2β and p53 was investigated by a Co-IP assay. Lysates were isolated from Hek293 cells cotransfected with HA-p53 and Myc-CCDC106 (**a**) and HeLa cells (**b**) were precipitated with control IgG or the indicated antibody. The immune complexes were subjected to WB. **c** Immunofluorescence staining of HeLa cells. A murine anti-CK2β monoclonal antibody and a Texas Red-conjugated anti-mouse IgG secondary antibody (red) were used to analyze CK2β, whereas a rabbit anti-CCDC106 polyclonal antibody and a FITC-conjugated anti-rabbit IgG secondary antibody (green) were used to analyze CCDC106. Nuclei were stained with Hoechst 33258 (blue). The yellow color in the merged image represents the colocalization of CK2β and the CCDC106 protein. **d** Phos-tag SDS-PAGE and WB analysis. The bacterially expressed and purified GST-fusion proteins were incubated with CK2 holoenzyme for 1 h. Then, the reaction mixture was separated by Phos-tag SDS-PAGE and subjected to WB using anti-GST antibody. **e** and **f** The influence of CK2β expression (**e**) and CX-4945 treatment (**f**) on the phosphorylation of the CCDC106 protein. HeLa cells were transfected with siRNA (siCK2β) and/or the expression plasmid (HA-CK2β) of CK2β (**e**) or treated with increasing concentrations of CX-4945 (**f**). At 24 h after transfection or treatment, phosphorylation of CCDC106 was analyzed by phospho-CCDC106 (p-S130) and phospho-CCDC106 (p-S147) antibodies

By searching the BioGRID interaction database (https://thebiogrid.org), we observed that CK2β had been identified to be a potential interacting partner of the CCDC106 protein by high-throughput affinity-purification mass spectrometry [29]. Thus, we performed a co-IP assay to confirm the interaction between CK2β and CCDC106. Lysates were prepared from HEK293 cells transiently transfected with Myc-CCDC106 and HA-CK2β plasmids and immunoprecipitated with anti-Myc antibody or control IgG. Western blot analysis of the coimmunoprecipitated products showed that HA-CK2β was present in the complex precipitated with the anti-Myc antibody but not in that with the control IgG (Fig. 2a). Further co-IP experiments with endogenous proteins from HeLa cells showed that the CCDC106 protein was recovered in a coimmunoprecipitated complex with the CK2β antibody (Fig. 2b). The abovementioned co-IP assays with both endogenous proteins and transiently expressed tagged proteins indicated that the CCDC106 protein interacted with CK2β. Their interaction was further validated by an immunofluorescence assay. In living HeLa cells, CCDC106 and CK2β were colocalized in the nuclei (Fig. 2c).

The CK2β subunit of protein kinase CK2 has been shown to be required for specific recognition of the substrate [12]. The abovementioned results suggested that CCDC106 was a potential substrate of CK2. The general recognition motif for phosphorylation by CK2 is S/T-X-X-D/E/pS [30]. We searched the database of eukaryotic linear motifs (ELM, http://elm.eu.org) and identified two putative CK2 phosphorylation sites in the CCDC106 protein, which included S130 and S147. To verify whether these two residues are the actual CK2 phosphorylation sites, we performed an in vitro phosphorylation assay using bacterially expressed wild-type and mutant CCDC106 proteins, and their phosphorylation status was investigated with the Phos-tag electrophoresis technique [24]. Accordingly, the regular CCDC106 protein was observed as only one band, whereas after incubation with CK2, CCDC106 showed two bands, the upper and lower bands represent phosphorylated and unphosphorylated CCDC106, respectively (Fig. 2d). Compared with the wild-type CCDC106 protein, substitution of S130 or S147 with Ala led to weakening of the phosphorylated bands and augmentation of the unphosphorylated bands, whereas substitution of both S130 and S147 completely abrogated the phosphorylated bands (Fig. 2d). This result indicated that both S130 and S147 can be phosphorylated by CK2.

Subsequently, phosphorylation-specific antibodies were used to validate the in vivo phosphorylation of CCDC106. As shown in Fig. 2e, knockdown of CK2β decreased the phosphorylation level of CCDC106 at S130 and S147 in HeLa cells, while overexpression of CK2β highly increased the level of phosphorylated CCDC106. Moreover, with increasing concentrations of the CK2 inhibitor CX-4945, the levels of phosphorylated CCDC106 dose-dependently decreased in HeLa cells (Fig. 2f, Additional file 1: Figure S3).

### Phosphorylation of CCDC106 at S130 and S147 is required for the interaction with p53 and localization of CCDC106, respectively

**Fig. 3:**
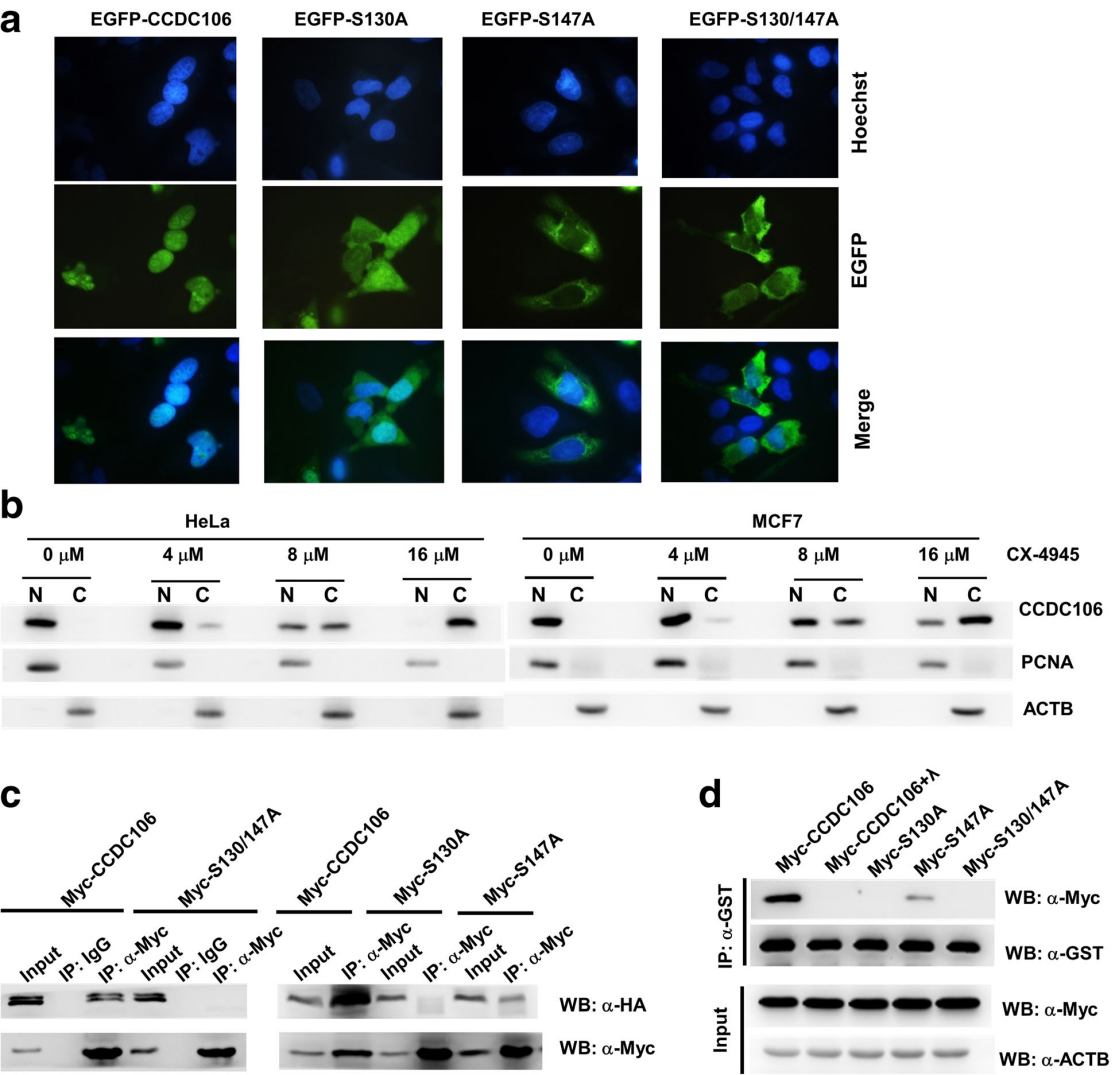
Phosphorylation of CCDC106 at S130 and S147 is required for CCDC106 interaction with p53. **a** The subcellular localization of wild-type and mutant EGFP-CCDC106 fusion proteins. The EGFP fusion protein-expressing plasmids were transfected into HeLa cells, and EGFP fluorescence was examined directly at 24 h posttransfection by fluorescence microscopy. Nuclei were stained with Hoechst 33258. Merge represents the combined image of EGFP fluorescence and nucleus staining. **b** The influence of CX-4945 treatment on the subcellular localization of the CCDC106 protein in HeLa and MCF7 cells. At 24 h after treatment with different concentrations of CX-4945, cell lysates were harvested, and the cytoplasmic (C) and nuclear (N) fractions were separated and subjected to WB. PCNA and ACTB were used as markers of nuclear and cytoplasmic proteins, respectively. **c** The interaction between p53 and wild-type or mutant p53 was analyzed by a Co-IP assay. HEK293 cells were transiently transfected with HA-p53 and Myc-tagged wild-type or mutant CCDC106 expression plasmids. Cell lysates (800 μg) were precipitated with an IgG or anti-Myc antibody, and the immune complexes were subjected to WB with an anti-HA or anti-Myc antibody. The input is equivalent to 10% of the lysate used for co-IP. **d** The interaction between p53 and wild-type or mutant p53 was detected by a GST pull-down assay. The bacterially expressed and purified GST-p53 fusion protein was incubated with the total lysates of HeLa cells transfected with a wild-type or mutant CCDC106 expression plasmid. GST-p53 was pulled down with glutathione agarose beads, and the associated Myc-CCDC106 fusion protein was analyzed by Western blotting with an antibody against Myc-tag. λ: cell lysate treated with λ protein phosphatase

CCDC106 is a nuclear protein that contains a nuclear localization signal (NLS) at residues 151–164 [9]. The CK2 phosphorylation sites of CCDC106 are near the NLS. Therefore, we investigated whether CK2 phosphorylation affects the subcellular distribution of CCDC106. EGFP-tagged wild-type or mutant CCDC106 expression plasmid was transfected into HeLa cells, and the EGFP fusion protein in living cells was analyzed by fluorescence microscopy. Consistent with our previous results [9], EGFP-CCDC106 was exclusively located in the nucleus. However, the mutant EGFP-S130A was distributed in both the cytoplasm and nucleus, while EGFP-S147A and EGFP-S130/147A were exclusively located in the cytoplasm (Fig. 3a). The result indicates that phosphorylation of CCDC106 at S147 and S130 is required for its nuclear localization. In particular, S147 had more influence on its nuclear localization than S130, presumably because S147 is closer to the position of NLS (^151^RRRQKQKGGASRRR^164^) [9] than S130. Additionally, treatment with the CK2 inhibitor CX-4945 decreased the nuclear distribution of endogenous CCDC106 in HeLa and MCF7 cells (Fig. 3b).

Accordingly, the influence of CCDC106 phosphorylation on the interaction between CCDC106 and p53 was analyzed using a co-IP assay. The wild-type CCDC106 could bind to p53, but the S130A mutant or S130/147A mutant lost the binding activity. Although the S147A mutant was still able to interact with p53, its interaction with p53 was weaker than that of wildtype CCDC106 (Fig. 3c). To eliminate the effects of subcellular localization on the interaction between CCDC106 and p53, we incubated the bacterially expressed and purified GST-p53 fusion protein with the total lysates of HeLa cells transfected with wild-type or mutant CCDC106 expression plasmids. GST-p53 was pulled down with glutathione agarose beads, and the associated Myc-CCDC106 fusion protein was analyzed by Western blotting with an antibody against Myc-tag. Consistent with the results of the co-IP assay, both wild-type CCDC106 and the S147A mutant could associate with p53, but S130A or the S130/147A mutant could not, and λ-phosphatase treatment abolished the interaction between wild-type CCDC106 and p53 (Fig. 3d).

The abovementioned results indicated that S130 phosphorylation is required for the interaction between CCDC106 and p53, while S147 phosphorylation mainly contributes to its nuclear localization.

### Inhibition of CCDC106 phosphorylation suppresses CCDC106-dependent degradation of the p53 protein

**Fig. 4:**
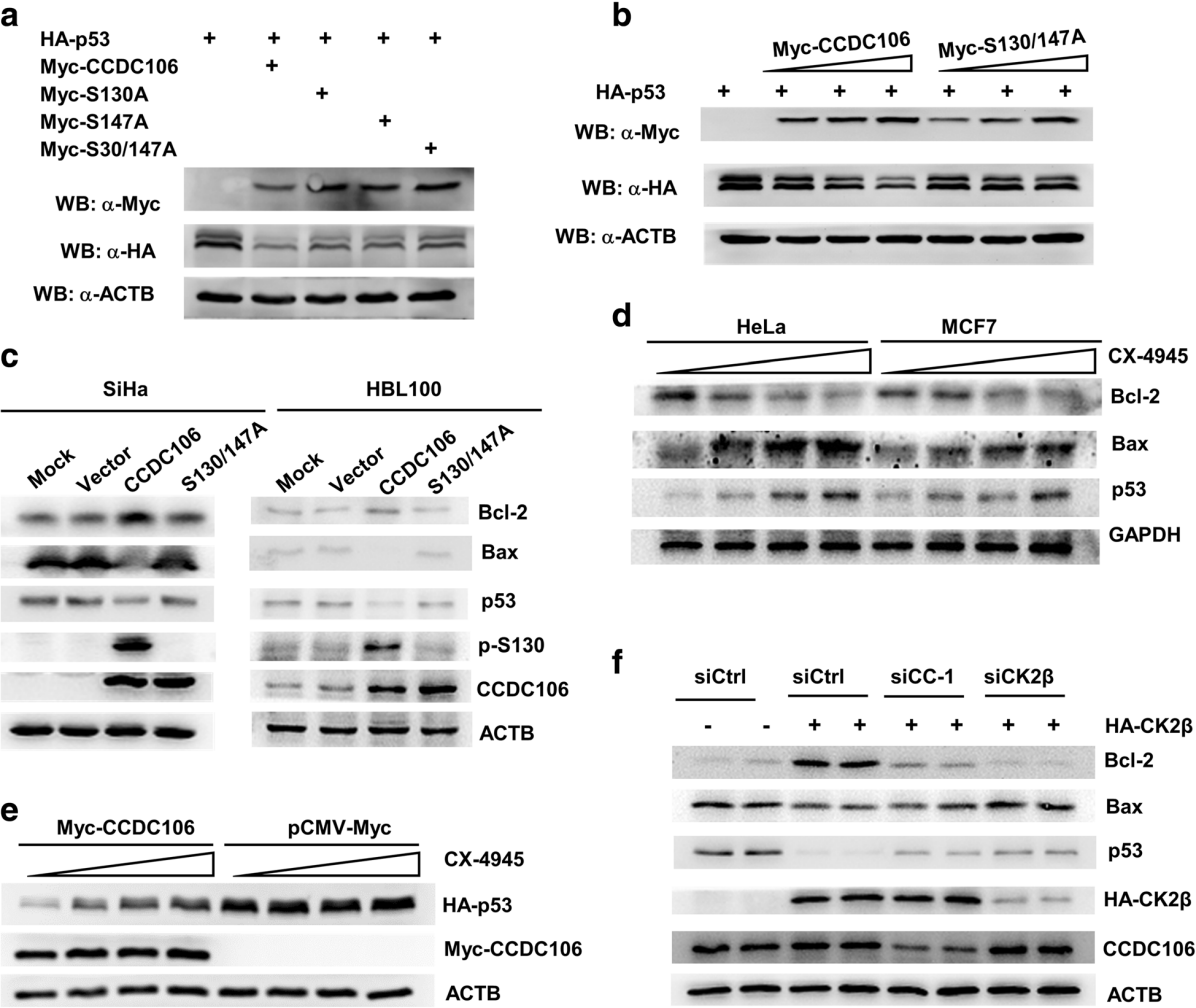
Inhibition of CCDC106 phosphorylation suppresses CCDC106-dependent degradation of the p53 protein. **a** and **b** The influence of wild-type and mutant CCDC106 on p53 protein expression levels. HA-p53 was cotransfected with an equal (**a**) or an increasing (**b**) amount of wild-type or mutant Myc-CCDC106 plasmid into HEK293 cells, and cell lysates were prepared and subjected to WB using anti-HA and anti-Myc antibodies at 24 h posttransfection. **c** The influence of wild-type and mutant CCDC106 on the expression levels of p53 and its targets. Cell lysates were extracted from SiHa and HBL100 cells stably expressing empty vector, CCDC106 or S130/147A mutant CCDC106, and subjected to WB. **d** The influence of CX-4945 treatment on the expression levels of p53 and its targets. Cells were treated with increasing concentrations of CX-4945 and harvested for WB analysis 24 h after treatment. **e** The influence of CX-4945 treatment on the expression of HA-p53 fusion protein. HA-p53 plasmid was cotransfected with empty vector or Myc-CCDC106 plasmid into HEK293 cells. Then, the cells were treated with CX-4945 for 24 h. HA-p53 fusion protein expression levels were analyzed by WB using an anti-HA antibody. **f** Silencing CCDC106 significantly reduces the effect of CK2β overexpression on p53 stability. HeLa cells were cotransfected with CK2β and siRNA targeting CCDC106 or CK2β, and harvested for WB analysis at 24 h posttransfection

To investigate the influence of phosphorylation on CCDC106-mediated degradation of the p53 protein, we cotransfected the p53 expression plasmid HA-p53 with the expression plasmid of wild-type or mutant CCDC106 into HEK293 cells and analyzed the level of the HA-p53 fusion protein at 24 h posttransfection. Consistent with our previous results [9], cotransfection with Myc-CCDC106 led to a reduction in HA-p53 levels, but transfection with Myc-S130A, Myc-S147A or the Myc-S130/147 mutant attenuated the HA-p53 degradation as compared to wildtype Myc-CCDC106 (Fig. 4a). Immunofluorescence staining also demonstrated that Myc-CCDC106, but not Myc-S130A or Myc-S147A, reduces the expression of endogenous p53 protein in HeLa (Additional file 1: Figure S4a, b and c). Moreover, with the increase of Myc-CCDC106 expression, the level of HA-p53 protein expression declined, but the HA-p53 protein expression level did not change in the cells transfected with Myc-S130A, Myc-S147A or Myc-S130/147A (Fig. 4b, Additional file 1: Figure S4d). Additionally, we compared the effects of wildtype and mutant CCDC106 on the expression of endogenous p53 and its targets in SiHa and HBL100 cells. The results showed that overexpression of CCDC106 reduced the expression levels of p53 and Bax but increased the level of Bcl2 expression, whereas overexpression of the S130/147A mutant had a slight effect on the expression levels of p53, Bax and Bcl2 (Fig. 4c). This result suggested that phosphorylation of CCDC106 by CK2 is required for CCDC106-mediated degradation of the p53 protein.

To confirm this result, we used the CK2 inhibitor CX-4945 to treat HeLa and MCF7. With the increase in CX-4945 concentration, the expression levels of p53 and Bax increased, while the expression levels of Bcl2 decreased in the cells (Fig. 4d). After treatment with CX-4945, the expression level of the HA-p53 fusion protein also increased in HEK293 cells cotransfected with HA-p53 and Myc-CCDC106, but the HA-p53 protein expression level was not affected by CX-4945 in HEK293 cells without Myc-CCDC106 transfection (Fig. 4e). These results indicated that CX-4945 increased the level of p53 protein expression by inhibiting the phosphorylation of CCDC106. However, silencing CCDC106 can significantly rescue p53 expression, but not completely abrogated the effect of CK2β overexpression on p53 stability (Fig. 4f), presumably because CK2 can also affect p53 stability through other pathways [20, 21].

### Inhibition of CCDC106 phosphorylation inhibits the oncogenicity function of CCDC106

**Fig. 5:**
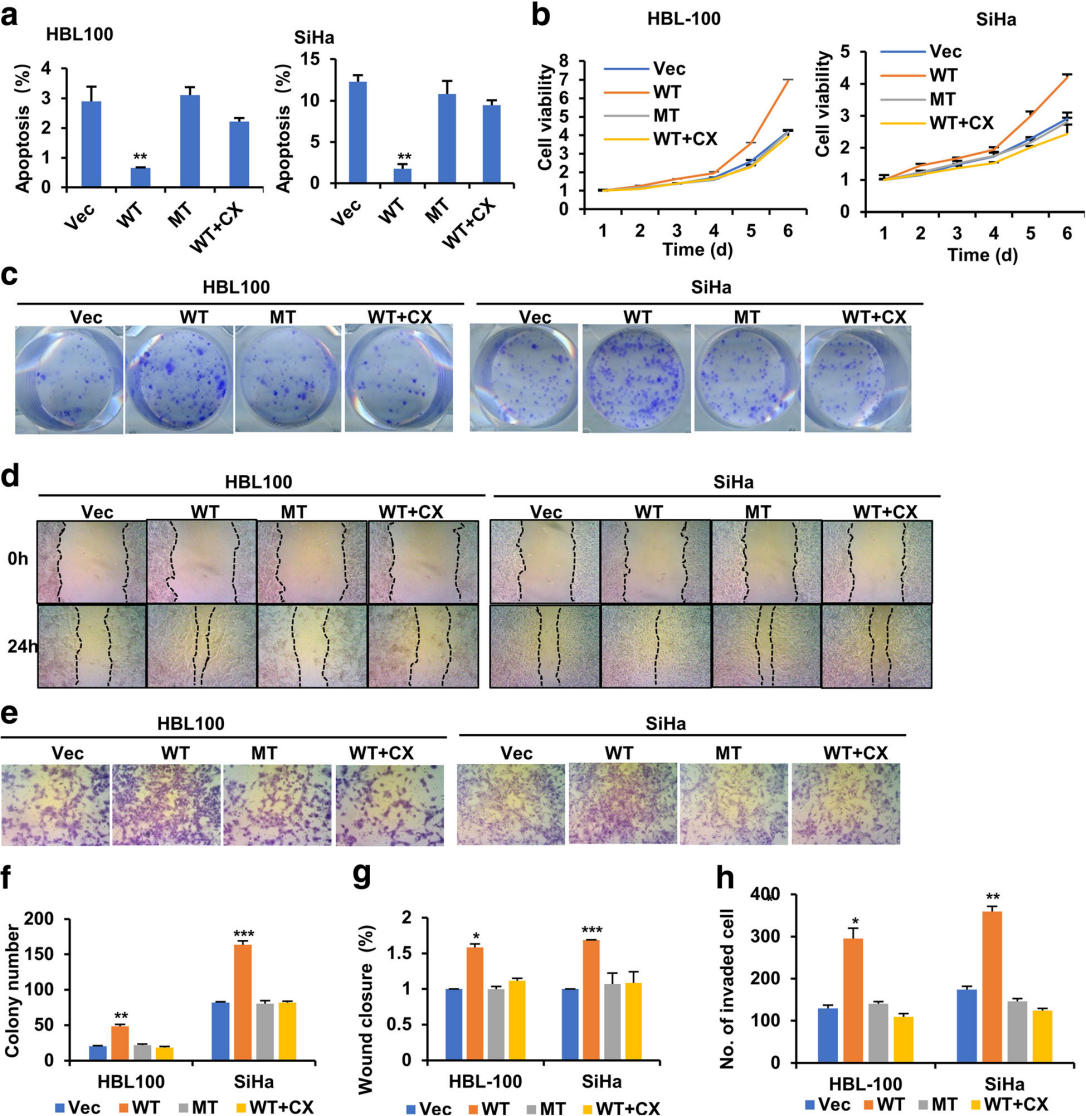
Inhibition of CCDC106 phosphorylation suppresses tumorigenesis and metastasis. a The apoptosis rates were investigated by annexin V-FITC/PI double staining and FCM analysis (FCM graphs are shown in Additional file 1: Figure S5). **b** Cell viability was determined by an MTT assay. **c** Representative images of cell colonies. **d** Representative images of a wound area at the indicated timepoints. **e** Representative images of invaded cells in the Transwell invasion assay. **f**, **g** and **h** Statistical analysis of colony numbers (**f**), the percentage of wound closure at 24 h after scratching (**g**) and the invaded cells in the Transwell invasion assay (**h**). All the values are presented as the means ± S.D. for at least three independent in vitro experiments. Differences between control and experimental groups were analyzed by Student’s t-test; *p-value ≤0.05, **p-value ≤0.01, and ***p-value ≤0.001. Vec, WT, and MT represent cells stably expressing empty vector, CCDC106 and S130/147A mutant, respectively; CX: CX-4945

**Fig. 6:**
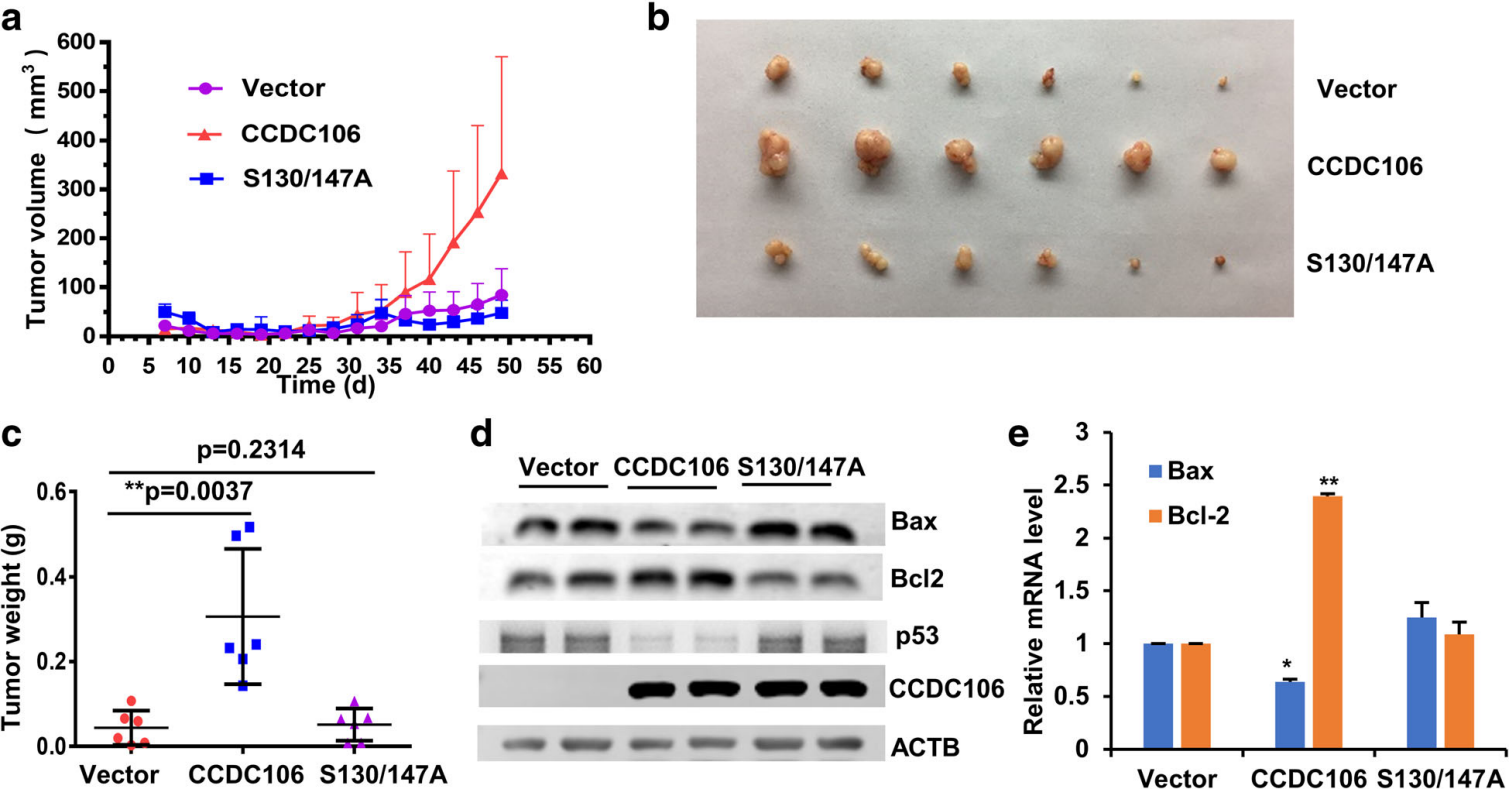
Overexpression of CCDC106, but not S130/147A mutant, enhances the growth of xenograft tumors. BALB/c nude mice were injected with 5 × 10^6^ SiHa cells expressing empty vector, CCDC106 or S130/147A mutant. **a** Dynamic volume (mean ± S.D., *n* = 6) of xenograft tumors at different timepoints after injection. **b** Images of the xenograft tumors at 49 d after injection. **c** Average weight (mean ± S.D., n = 6) of xenograft tumors at 49 d after injection. **d** WB analysis of the expression of Bax, Bcl2, p53 and CCDC106 in tumors. **e** Real-time PCR analysis of the mRNA expression levels of Bcl2 and Bax in tumors. Differences between control and experimental groups were analyzed by Student’s t-test; *p-value ≤0.05, **p-value ≤0.01, and ***p-value ≤0.001

Next, we further investigated the effects of CCDC106 overexpression. Contrary to the effects of CCDC106 knockdown, overexpression of CCDC106 in HBL100 and SiHa cells suppressed apoptosis (Fig. 5a, Additional file 1: Figure S5) and enhanced cell viability, migration and invasion (Fig. 5b-h). Substitution of both S130 and S147 with Ala or treatment with CX4945 significantly suppressed the effects of CCDC106 on cell behaviors (Fig. 5a-h). However, overexpression of wild-type or mutant CCDC106 had no effect on the behavior of p53-mutant cells (MDA-MB-231) (Additional file 1: Figure S6). These results indicated that phosphorylation by CK2 enhances the oncogenic function of CCDC106 in a p53-dependent manner. Additionally, we further investigated the in vivo effects of CCDC106 on tumor growth using a subcutaneous xenograft tumor mouse model. The growth rate and weight of tumors derived from CCDC106-overexpressed SiHa cells are significantly higher than those of tumors derived from control cells, but the tumors derived from S130/147A-overexpressed SiHa cells had no significant difference in growth rate and weight with the tumors derived from control cells (Fig. 6a-c). Analysis of tumors by Western blotting and realtime PCR revealed that overexpression of CCDC106 decreased the expression of p53 and Bax but increased Bcl2 expression. However, overexpression of S130/147A mutant had no significant influences on the expression of p53, Bax and Bcl2 (Fig. 6d-e).

### CCDC106 increases CX-4945 resistance of cancer cells with wtp53

**Fig. 7:**
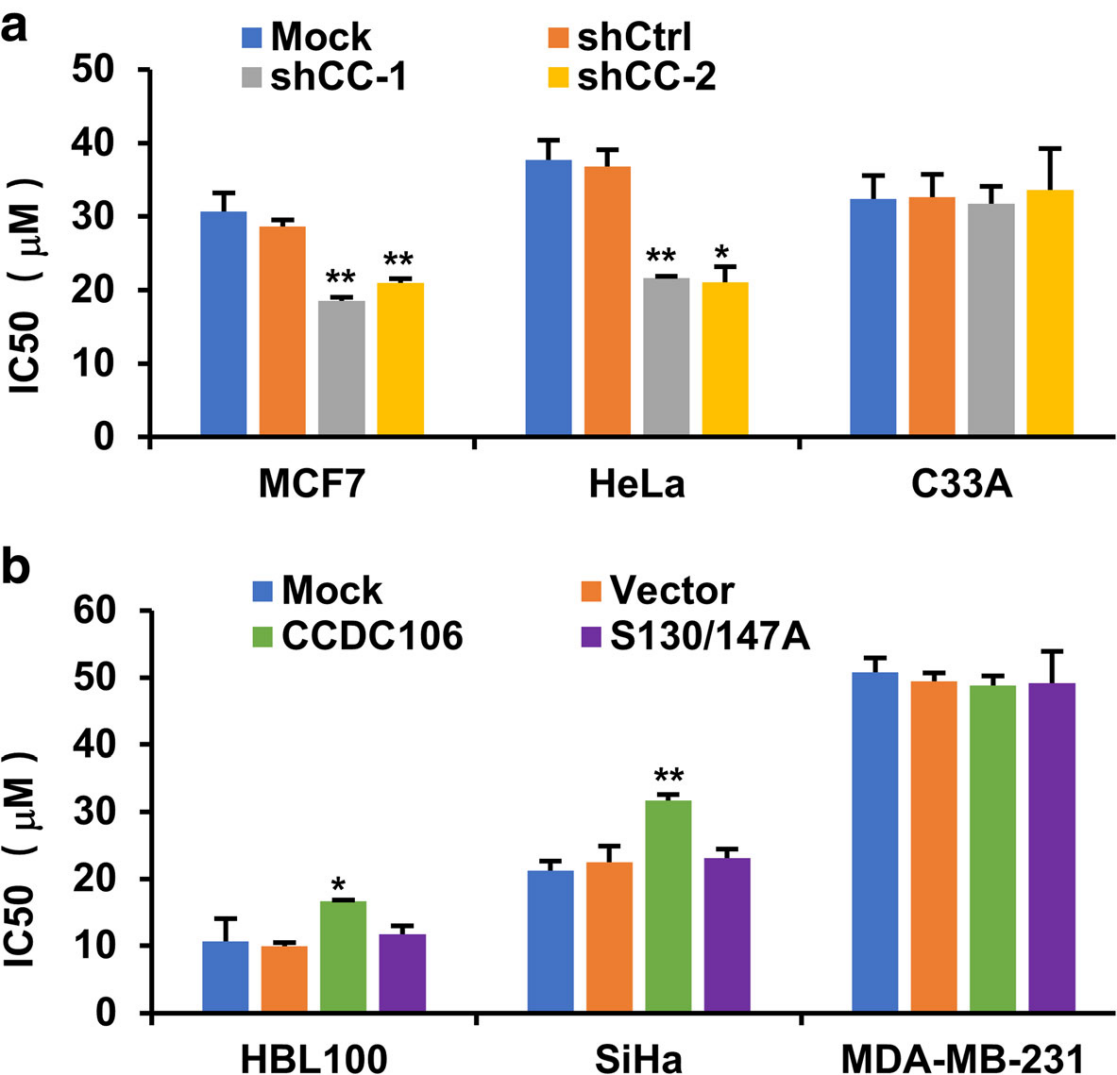
CCDC106 enhances CX-4945 resistance of cancer cells in a p53-dependent manner. **a** Silencing CCDC106 decreases IC50 values in MCF7 and HeLa cells with wtp53, but not in C33A with mtp53. **b** CCDC106 overexpression increases IC50 values in HBL100 and SiHa cells with wtp53, but not in MDA-MB-231 cells with mtp53. Cells were seeded in 96-well plates and treated with different concentrations of CX-4945 for 24 h. Then, cell viability was determined by an MTT assay. Based on the results of the MTT assay, the IC50 was calculated. All the values are presented as the means ± S.D. for at least three independent in vitro experiments. Differences between control and experimental groups were analyzed by Student’s t-test; *p-value ≤0.05, **p-value ≤0.01, and ***p-value ≤0.001

The abovementioned results demonstrated that CX-4945 can exert antitumor roles by inhibiting the phosphorylation of CCDC106. We further examined the CCDC106 status on sensitivity to CX-4945 treatment and observed that knockdown of CCDC106 increased the sensitivity to CX-4945 in MCF7 and HeLa cells (Fig. 7a). Conversely, overexpression of CCDC106 reduced the sensitivity to CX-4945 in HBL100 and SiHa cells (Fig. 7b). Moreover, mutation of the phosphorylated site in CCDC106 facilitated the sensitivity to CX-4946 in HBL100 and SiHa cells (Fig. 7b). However, the sensitivity to CX-4945 was observed to be independent of the CCDC106 status in the cells with mtp53 (C33A and MDA-MB-231 cells) (Fig. 7).

## Discussion

We previously showed that CCDC106 negatively regulates p53 stability [9]. In this study, we further confirmed that CCDC106 promotes p53 degradation by facilitating its polyubiquitination in breast and cervical cancer cells (MCF7 and HeLa cells, respectively). Knockdown of CCDC106 in both MCF7 and HeLa cells increased the expression levels of proapoptotic p53 and Bax and decreased the expression levels of antiapoptotic Bcl2, leading to a rise of cell apoptosis. Consistently, suppression of CCDC106 inhibits colony formation, migration and invasion in cancer cells (MCF7 and HeLa cells) with wtp53. Conversely, overexpression of CCDC106 showed opposite effects in HBL100 and SiHa cells. Actually, CCDC106 had no influence on the stability of mtp53 and the behavior of the cells with mtp53 (MDA-MB-231 and C33A cells). These results suggested that CCDC106 exerts oncogenic roles in a p53-dependent manner. This finding is not consistent with a report by Zhang X et al. that suggested that the oncogenic role of CCDC106 is independent of p53 status in NSCLC cells [10]. The different effects of CCDC106 in different cancer cells may result from its different subcellular localization. Based on the observations by Zhang X et al., CCDC106 is localized in the cytoplasm in A549 lung cancer cells [10], while p53 is mainly distributed in the nuclei; therefore, CCDC106 cannot interact with p53 in A549 cells. However, the present study showed that CCDC106 interacts with p53 in nuclei of HeLa and MCF7 cells.

It has been shown that CK2 can indirectly regulate p53 stability by activating the E3 ubiquitin ligases MDM2 [20] or NAD-dependent deacetylase SIRT1 [21]. In this study, we discovered a new mode of regulating p53 stability by CK2. Based on our results, CK2-mediated phosphorylation of CCDC106 is required for its nuclear localization and interaction with p53, facilitating the degradation of p53. Inhibiting CCDC106 phosphorylation by substituting both S130 and S147 with alanine or treating cells with the CK2 inhibitor CX4945 abrogated CCDC106-induced p53 degradation and its oncogenic function in breast and cervical cancer cells. These results indicated that CK2 promotes the degradation of p53 by phosphorylating and activating CCDC106. Moreover, we showed that the inhibition of CCDC106 sensitizes breast and cervical cancer cells to CX-4945. This finding is very important since CK2 activity is elevated in breast and cervical cancers [15, 16], and inhibition of CK2 is a promising treatment for both cancer types [17, 18]. Our results indicate that combinational use of a CCDC106 siRNA and a CK2 inhibitor is a useful therapeutic strategy for breast and cervical cancers expressing wtp53. However, the beneficial effect of CCDC106 siRNA on the chemosensitivity to CX-4945 needs to be further confirmed in animals in vivo. It would also certainly be worth investigating CCDC106 expression in clinical samples of breast and cervical cancers to determine its clinicopathologic significance.

## Conclusions

In summary, we demonstrated that CCDC106 inhibits p53-mediated apoptosis, leading to the progression of breast and cervical cancer with wtp53, while its phosphorylation by CK2 is required for its interaction with p53 and oncogenic function. This study identified a CK2/CCDC106/p53 signaling axis in cancer progression, which may represent a new therapeutic target for cancer treatment.

## Additional files

Additional file 1: **Figure S1.** Knockdown of CCDC106 enhances cell apoptosis in the cells with wtp53 (HeLa, MCF7), but not the cells with mtp53 (C33A). **Figure S2.** Knockdown of CCDC106 does not affect p53 stability, apoptosis, growth, migration and invasion of C33A cells with mtp53. **Figure S3.** CX-4945 does not affect the expression of CK2α and CK2β. **Figure S4.** Mutation of S130 or S147 of CCDC106 protein suppresses CCDC106-dependent degradation of the p53 protein. **Figure S5.** Overexpression of CCDC106, but not mutant CCDC106, reduces cell apoptosis in the cells with wtp53 (HBL100, SiHa), but not the cells with mtp53 (MDA-MB-231). **Figure S6.** Overexpression of CCDC106 does not affect p53 stability, apoptosis, growth, migration and invasion of MDA-MB-231 cells with mtp53. (DOC 18895 kb)

## Abbreviations

BAX: BCL2 associated X protein
BCL2: B-cell lymphoma 2
CCDC106: Coiled-coil domain-containing protein 106
Co-IP: Co-immunoprecipitation
mtp53: Mutant p53
PAGE: Polyacrylamide gel electrophoresis
WB: Western blotting
wtp53: Wildtype p53
