# Supplementary material for: CK2-mediated CCDC106 phosphorylation is required for p53 degradation in cancer progression
Source: J Exp Clin Cancer Res. 2019 Mar 18;38:131. doi: 10.1186/s13046-019-1137-8 (PMC6423756; doi:10.1186/s13046-019-1137-8)
Supplement: Supplementary file 1 — Figure S1. Knockdown of CCDC106 enhances cell apoptosis in the cells with wtp53 (HeLa, MCF7), but not the cells with mtp53 (C33A). Figure S2. Knockdown of CCDC106 does not affect p53 stability, apoptosis, growth, migration and invasion of C33A cells with mtp53. Figure S3. CX-4945 does not affect the expression of CK2α and CK2β. Figure S4. Mutation of S130 or S147 of CCDC106 protein suppresses CCDC106-dependent degradation of the p53 protein. Figure S5. Overexpression of CCDC106, but not mutant CCDC106, reduces cell apoptosis in the cells with wtp53 (HBL100, SiHa), but not the cells with mtp53 (MDA-MB-231). Figure S6. Overexpression of CCDC106 does not affect p53 stability, apoptosis, growth, migration and invasion of MDA-MB-231 cells with mtp53. (DOC 18895 kb) [file 13046_2019_1137_MOESM1_ESM.doc]

**Supplementary Materials**

**
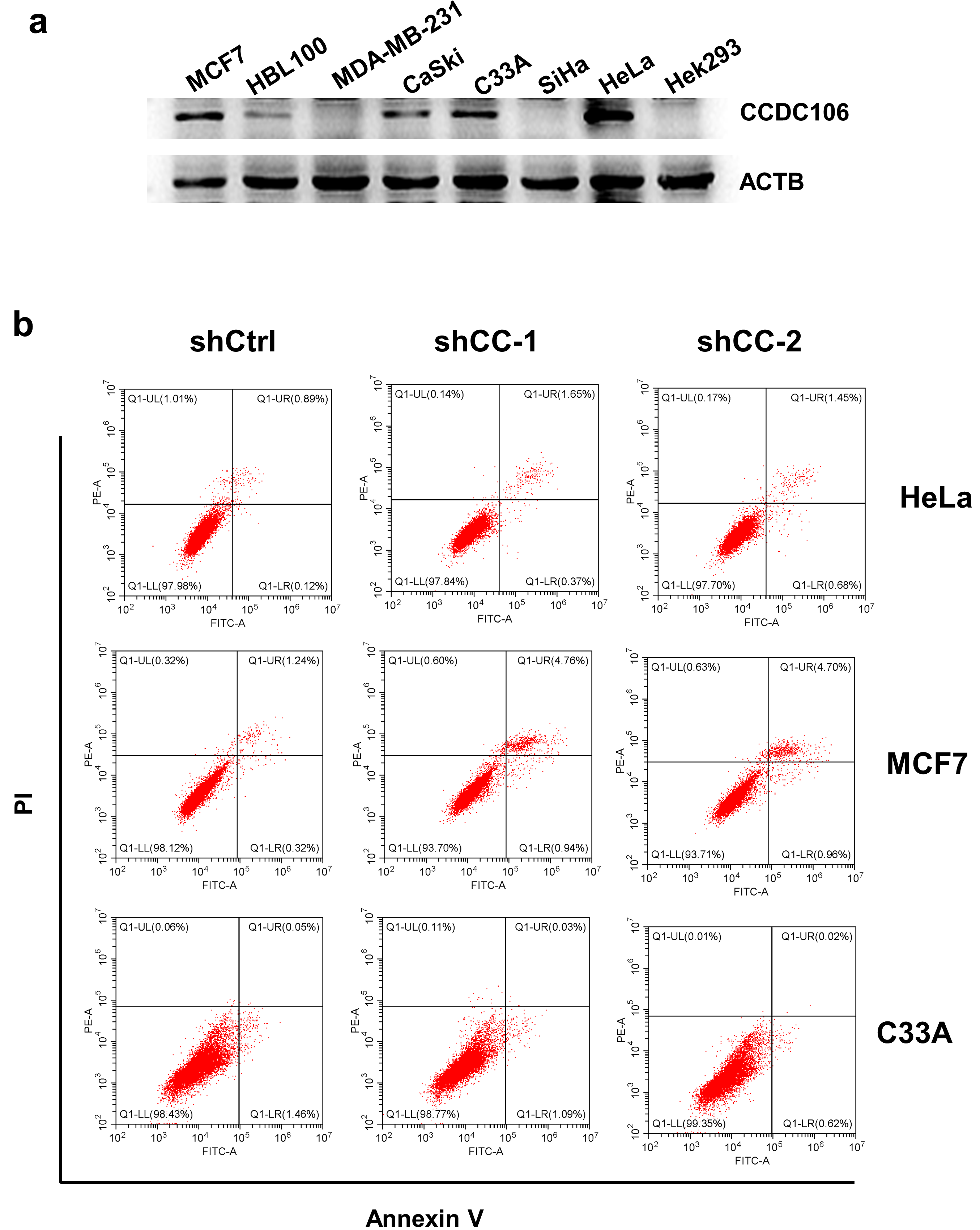
**

**Fig. S1** Knockdown of CCDC106 enhances cell apoptosis in the cells with wtp53 (HeLa, MCF7), but not the cells with mtp53 (C33A). **a** Expression of CCDC106 in some cell lines was detected by Western blotting (WB)**. b** Representative scatterplots of flow cytometry. Cells were sequentially stained with annexin V-FITC and propidium iodide (PI) (Invitrogen, Carlsbad, CA, USA) and then analyzed by flow cytometer (Beckman Coulter, Brea CA, USA).

[**英文Snomed词典**](javascript:void 0;)

[**英文Mesh词典**](javascript:void 0;)

[**医学详解词典**](javascript:void 0;)

[更多诊疗知识库](javascript:moredisease();)

[**疾病关联词典**](javascript:void 0;)

[**药品相关信息**](javascript:void 0;)

[更多相关药物](javascript:moredrug();)

[**英汉药名关联词典**](javascript:void 0;)

[**汉英药名关联词典**](javascript:void 0;)

[**中医主题词词典**](javascript:void 0;)

[**中医术语详解词典**](javascript:void 0;)

[**英汉人名地名词典**](javascript:void 0;)

[**汉英人名地名词典**](javascript:void 0;)

[**英汉医学缩略语词典**](javascript:void 0;)

[**汉英医学缩略语词典**](javascript:void 0;)

[同义词](javascript:void 0;) [反义词](javascript:void 0;) [英汉医学短语词典](javascript:void 0;) [英汉通用短语词典](javascript:void 0;)

**
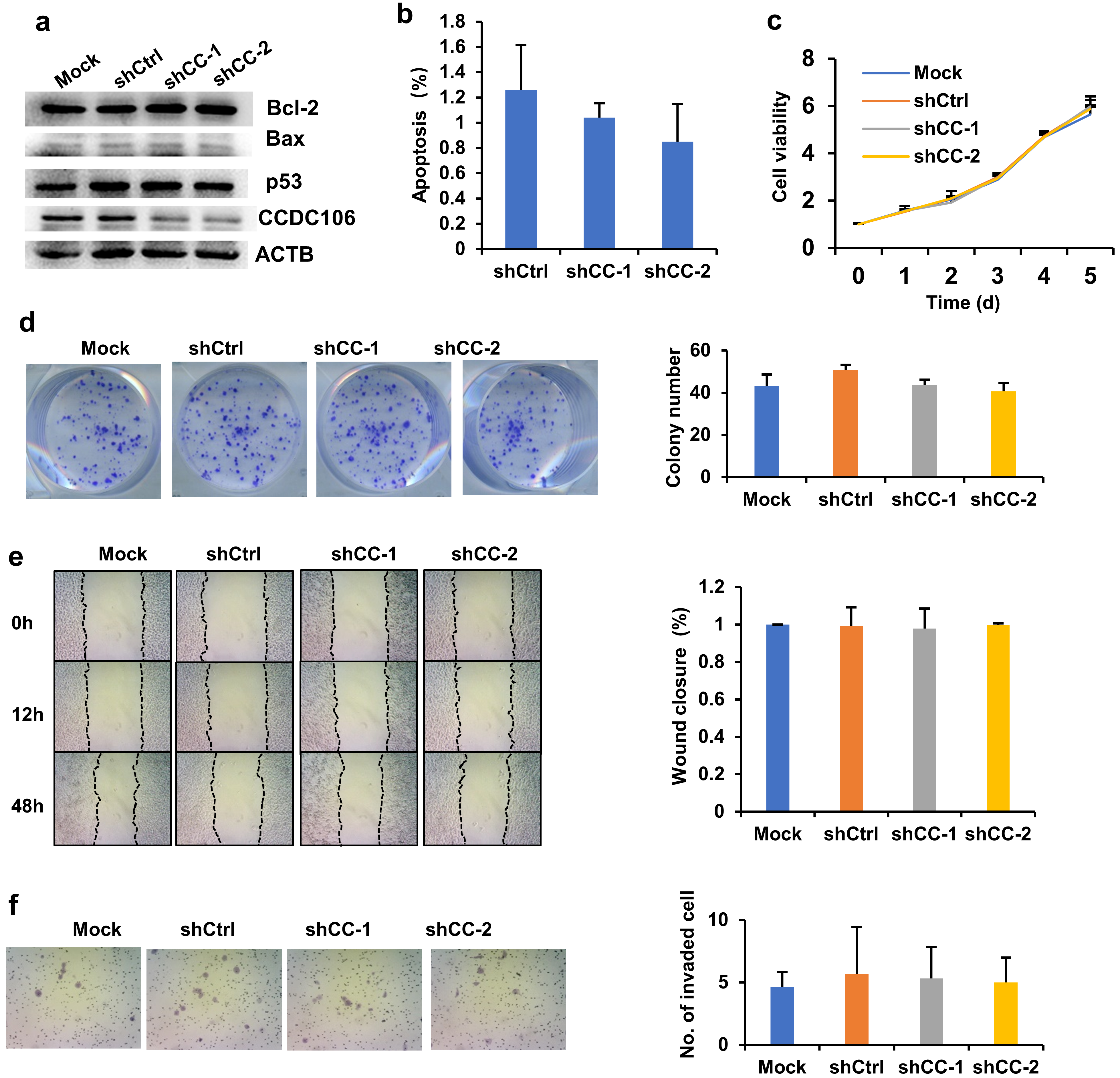
**

**Fig. S2** Knockdown of CCDC106 does not affect p53 stability, apoptosis, growth, migration and invasion of C33A cells with mtp53. **a** Western blotting analysis of CCDC106, p53 and the targets of p53. **b** The apoptosis rate was detected by Annexin V-FITC/PI double staining and flow cytometry. The representative graphs are shown in Figure S1B. **c** The cell viability was determined by MTT assay. **d** Representative images of colony (left panel) and statistical analysis of the colony numbers (right panel). **e** Representative images (left panel) of wound area at the indicated time and percentage of wound closure at 48 h after scratching (right panel). **f** Representative images (left panel) and statistical analysis (right panel) of invaded cells in the Transwell invasion assay. All the values are presented as means + S.D for at least three independent experiments. Differences between control and experiment groups were analyzed by student t-test; *p-value ˂ 0.05, **p-value ˂ 0.01.

**
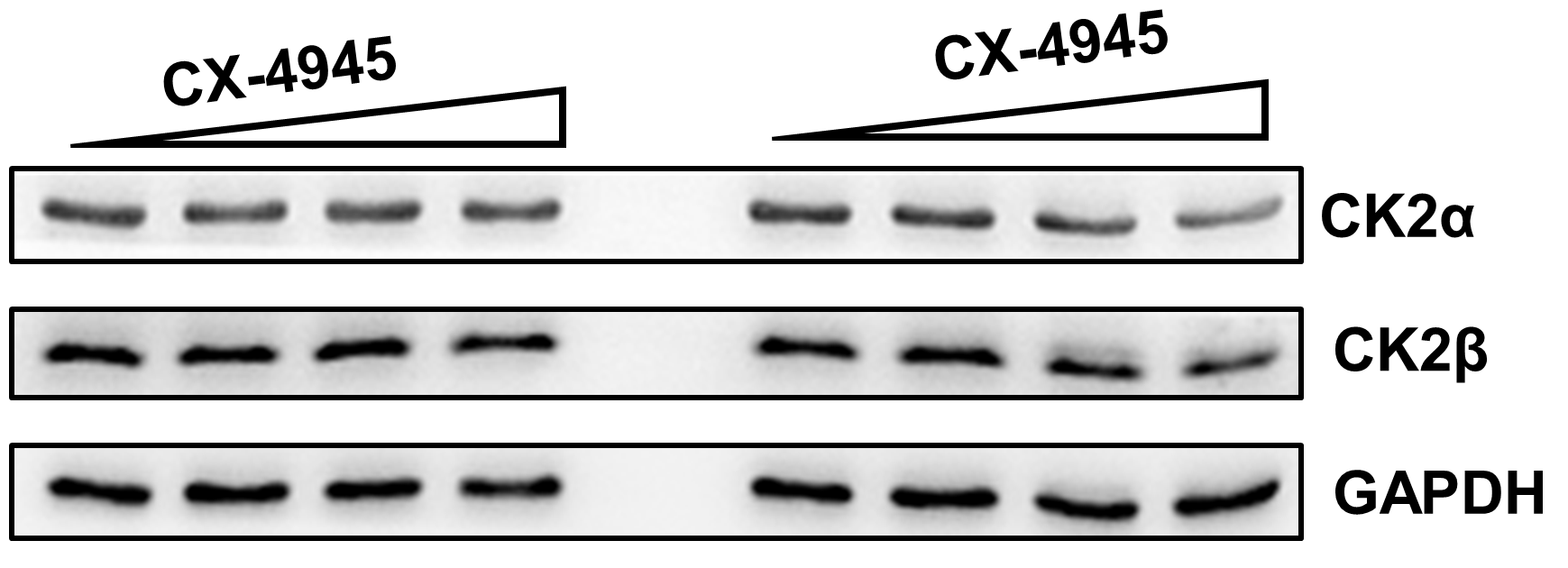
**

**Fig. S3** CX-4945 does not affect the expression of CK2α and CK2β. HeLa cells were treated with increasing concentrations of CX-4945. At 24 h after treatment, the levels of CK2α and CK2β proteins were analyzed by WB.

**
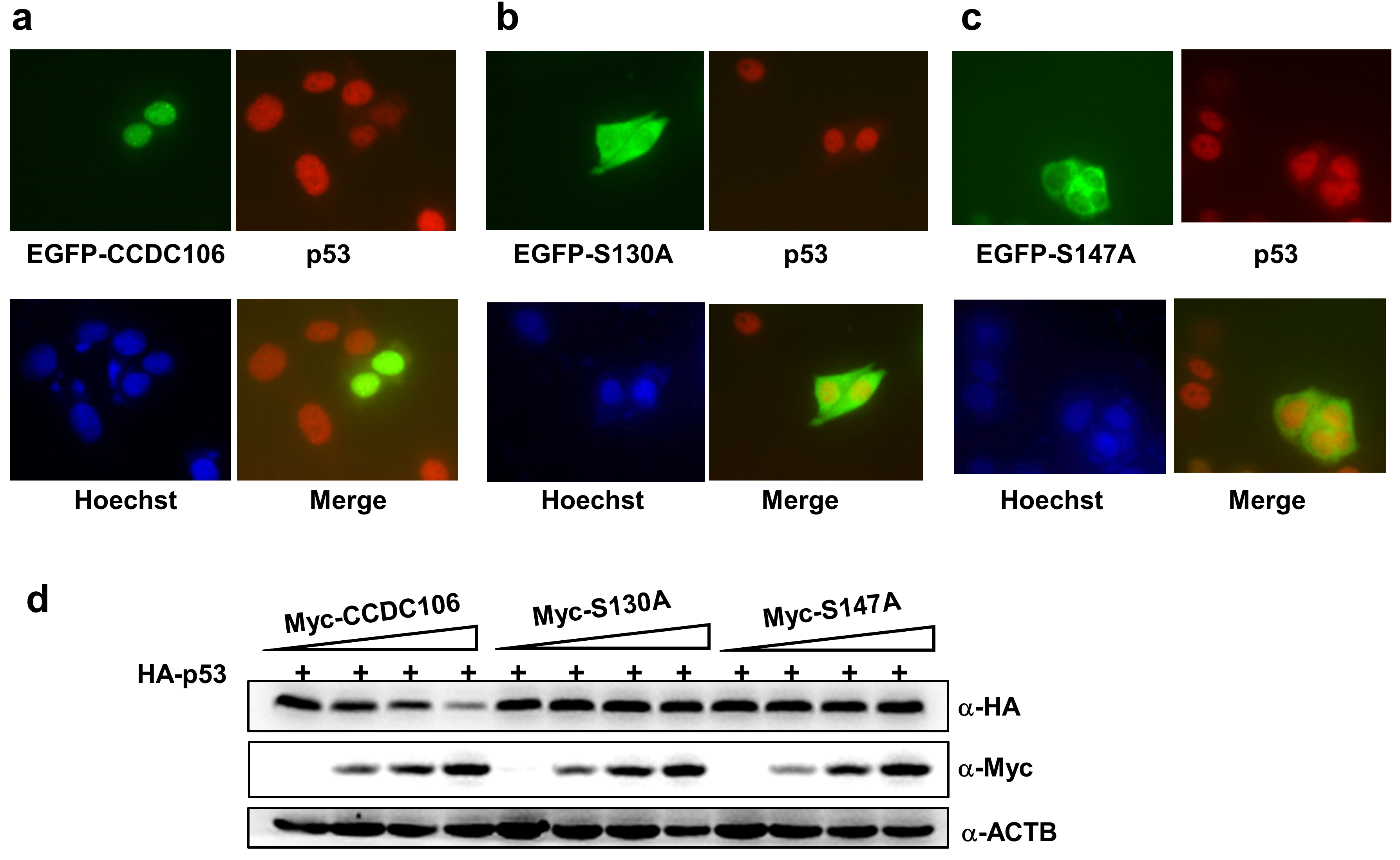
**

**Fig. S4** Mutation of S130 or S147 of CCDC106 protein suppresses CCDC106-dependent degradation of the p53 protein. **a**, **b** and **c** Overexpression of CCDC106, but not S130A or S147 mutant, reduces p53 staining. HeLa cells were transfected with EGFP-CCDC106 (**a**), EGFP-S130A (**b**) or EGFP-S147A (**c**). EGFP fusion proteins were excited with 488 nm light. The endogenous p53 protein was stained with a murine anti-p53 monoclonal antibody and a Texas Red-conjugated anti-mouse IgG secondary antibody. Nuclei were stained with Hoechst 33258. Merge represents the combined image of EGFP fluorescence and p53 staining. **d** Overexpression of CCDC106, but not S130A or S147 mutant, reduces p53 levels. HA-p53 was cotransfected with an increasing amount of wild-type or mutant CCDC106 plasmid into HEK293 cells, and cell lysates were subjected to WB using anti-HA and anti-Myc antibodies at 24 h posttransfection.

**
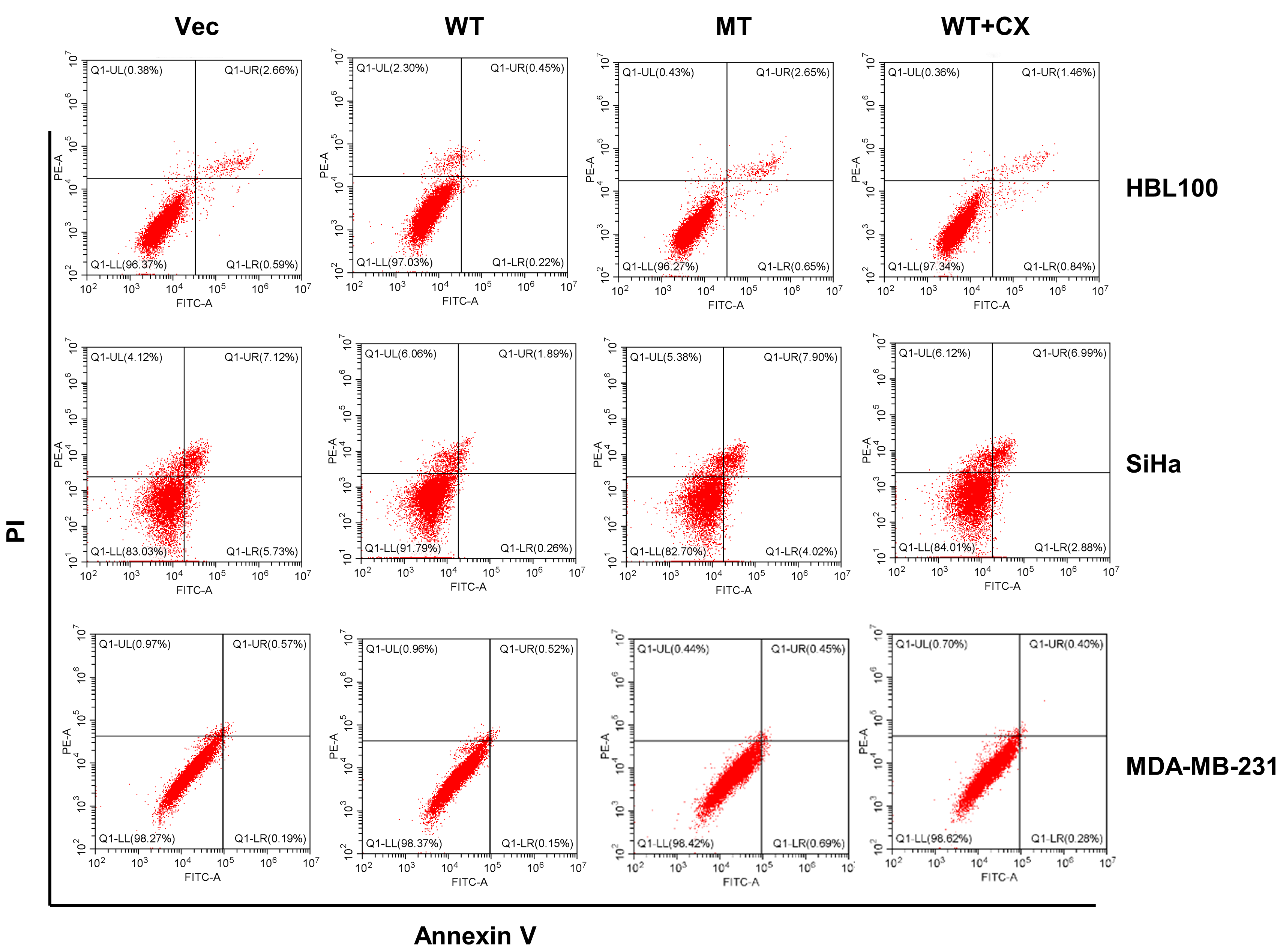
**

**Fig. S5** Overexpression of CCDC106, but not mutant CCDC106, reduces cell apoptosis in the cells with wtp53 (HBL100, SiHa), but not the cells with mtp53 (MDA-MB-231). Representative scatterplots of flow cytometry. Cells were sequentially stained with annexin V-FITC and propidium iodide (PI) and then analyzed by flow cytometer. The statistical analysis of three independent experiments are shown in Fig. 5a and Fig. S6.

**
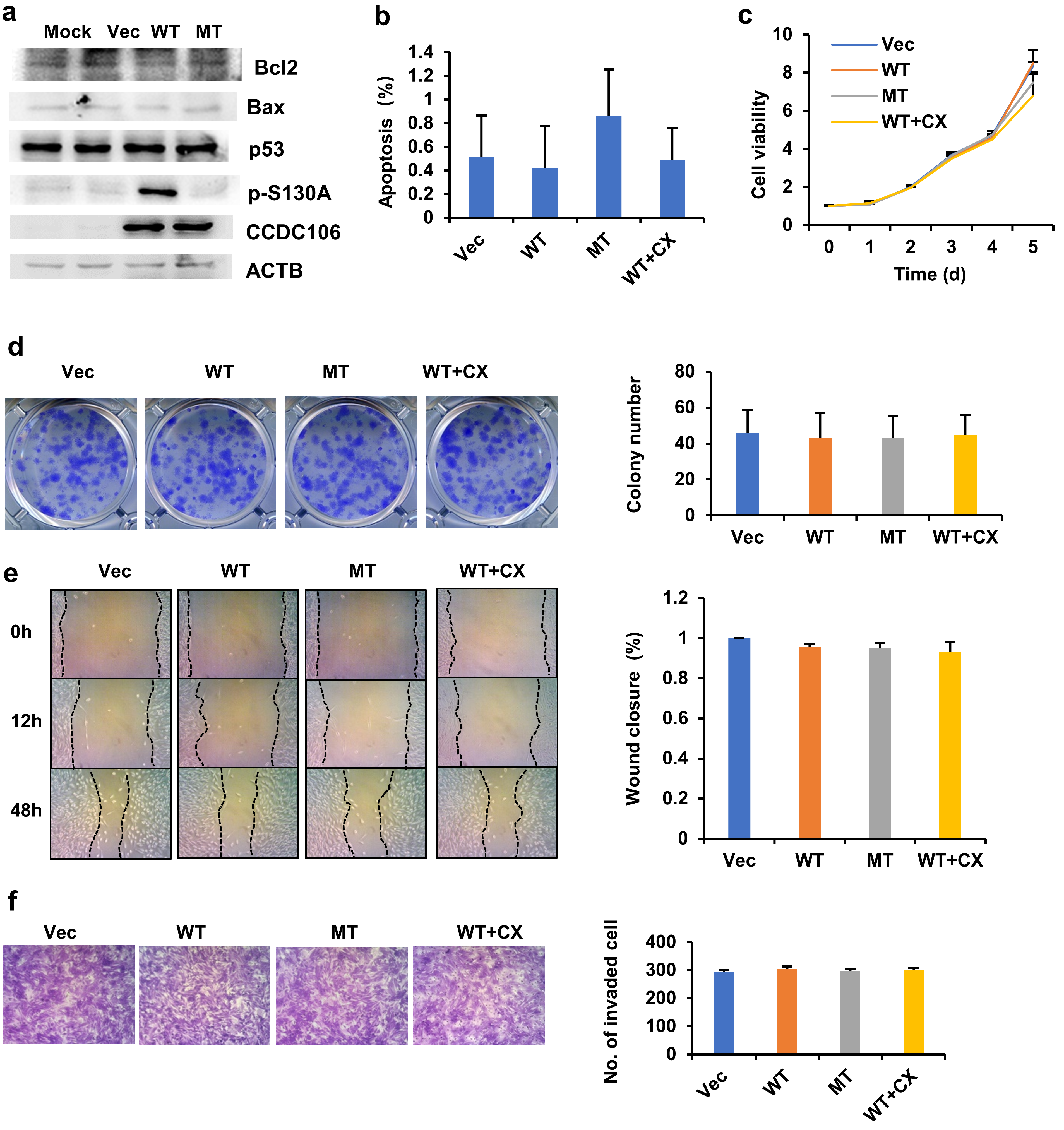
**

**Fig. S6** Overexpression of CCDC106 does not affect p53 stability, apoptosis, growth, migration and invasion of MDA-MB-231 cells with mtp53. **a** WB analysis of CCDC106, p53 and the targets of p53. **b** The apoptosis rate was detected by Annexin V-FITC/PI double staining and flow cytometry. **c** The cell viability was determined by MTT assay. **d** Representative images of colony (left panel) and statistical analysis of the colony numbers (right panel). **e** Representative images (left panel) of wound area at the indicated time and percentage of wound closure at 48 h after scratching (right panel). **f** Representative images (left panel) and statistical analysis (right panel) of invaded cells in the Transwell invasion assay. All the values are presented as means ± S.D for at least three independent experiments. Differences between control and experimental groups were analyzed by student t-test. Vec, WT, and MT represent cells stably expressing empty vector, CCDC106 and S130/147A mutant, respectively; CX: CX-4945. Differences between control and experiment groups were analyzed by student t-test; *p-value ≤ 0.05, **p-value ≤ 0.01.
